# Intraocular Pressure Values using IcareⓇ Rebound Tonometer and Correlation with Postconceptional Age in Premature Infants

**DOI:** 10.18502/jovr.v18i3.13774

**Published:** 2023-07-28

**Authors:** Catarina Monteiro, Maria Vivas, Júlio Almeida, Mário Ramalho, Mafalda Mota, Susana Teixeira, Isabel Prieto

**Affiliations:** ^1^Hospital Professor Doutor Fernando da Fonseca, Amadora, Portugal

**Keywords:** Intraocular Pressure, Postconceptional Age, Premature Infants, Rebound Tonometry

## Abstract

**Purpose:**

This study aimedto determine a normative range of intraocular pressure (IOP) values measured with Icare rebound tonometer in premature infants and evaluate IOP variation over time and its correlation with the progression of postconceptional age (PCA). By doing so, we also evaluated advantages of this IOP-measuring method in this population when compared to more traditional methods.

**Methods:**

We conducted a single-center prospective study that included premature infants (gestational age ≤ 32 weeks) who were admitted to the neonatal intensive care unit (NICU) in Hospital Professor Doutor Fernando Fonseca. The study took place between January and December 2021. IOP was measured using Icare tonometer on the occasion of the first retinopathy of prematurity (ROP) screening requested by the NICU and again after a two-week interval if PCA was still ≤ 37 weeks. IOP measurements were stopped at 37 weeks or if the infant was discharged. The evaluated outcomes were mean IOP values and their correlation with PCA.

**Results:**

Thirty-four eyes of 17 preterm infants with a mean gestational age of 29.4 ± 2.3 weeks and a mean birth weight of 1222.9 ± 361.9 gr were evaluated. The mean IOP registered was 16.1 ± 6.4 mmHg, with a median value of 15.3 mmHg. The top 90 ${\;}^{th}$ percentile was 22.1 mmHg and the bottom 10 ${\;}^{th}$ percentile was 9.0 mmHg. The average IOP reduction was 4.8 ± 6.7 mmHg (*P* = 0.0019) within the two-week interval of PCA.

**Conclusion:**

The mean IOP in premature infants was 16.1 ± 6.4 mmHg and this value significantly decreased by 4.8 ± 6.7 mmHg every two weeks of PCA.

##  INTRODUCTION

In order to detect developmental abnormalities and congenital diseases in the eye, it is crucial to have a good understanding of normal intraocular pressure (IOP) values and their changes over time in premature infants.^[1]^ The characteristics of the anterior chamber angle in the premature infant differ from that in adults, and parameters such as anterior chamber depth, trabecular-iris angle and iris thickness seem to show a linear relation with postconceptional age (PCA).^[2]^ To this date and to the best of our knowledge, very few studies have reported IOP in preterm infants, and with the advent of new handheld tonometers such as the iCare, measuring IOP could become part of the routine evaluation of these children. The purpose of this prospective longitudinal study is to determine average IOP values for premature infants and to evaluate their progression over time.

##  METHODS

This single-centerprospectivestudy included preterm infants with either gestational ages between 24 and 32 weeks or a birth weight of < 1500 gr, who were evaluated by the ophthalmology department as part of the routine retinopathy of prematurity (ROP) screening program. Children with chromosomal abnormalities and dysmorphic syndromes, congenital ocular abnormalities and children under invasive ventilation were excluded from the study. We included some eyes with early stages of ROP (stages I and II) but excluded all eyes with ROP in more advanced stages and those eyes submitted to laser therapy or anti-VEGF treatment. The study was conducted in the neonatal intensive care unit (NICU) of our hospital and all infants were examined while still hospitalized. PCA was defined as the gestational age plus the number of weeks since birth. Gestational age of the infants was determined based on obstetric history and first-trimester obstetric ultrasound.

Two experienced ophthalmologists specialized in pediatric ophthalmology performed the ocular examination in all studied infants. If the infant was irritable or crying, IOP measurement was postponed until the child was calm to avoid a Valsalva-like effect. No sedative drugs or muscle relaxants were given to the infants immediately before or during the examination, as these may have artificially lowered the IOP values. The evaluation was only performed if the child was clinically stable. The infant was laid on his/her back in the incubator and IOP was measured with an iCareⓇ IC200 rebound tonometer, without the use of topical anesthesia. The right eye was the first to be examined in all cases and measurements were obtained between 9 and 12 am. Three IOP measurements were taken for each eye and average IOP was calculated. IOP measurements were repeated every 2 weeks and stopped after 37 weeks of PCA or if the infant was discharged.

The study protocol was reviewed and approved by the Ethics Committee of the Hospital Professor Doutor Fernando da Fonseca, Amadora, Portugal under approval number 39/2022. The protocol of the study complied with the guidelines for human studies and the World Medical Association Declaration of Helsinki.

##  RESULTS

This study included 34 eyes from 17 premature infants, 26 males and 8 females, with a mean gestational age of 29.4 ± 2.3 weeks (24.9–32.7 weeks) and a mean birth weight of 1222.9 ± 361.9 gr. As there was no statistically significant difference between right and left eyes (*P* = 0.8), the results were analyzed together. The mean PCA was 33.9 ± 1.6 weeks with a range of 32 to 41 weeks. Twelve patients underwent two evaluations and ten were only submitted to one observation. Of the 34 included eyes, 6 had ROP stage I or stage II; none of these eyes were submitted to laser or anti-VEGF therapy.

**Figure 1:**
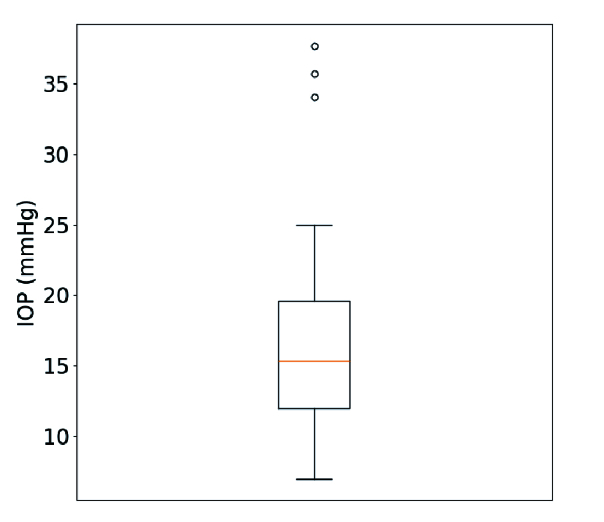
Box and whisker plot of IOP values. The box extends from the lower to the upper quartile values of the data (P25–P75) with a line at the median. The whiskers extend from the box to show the range of the data. Beyond the whiskers, data is considered as outliers and is plotted as individual points.

At the end of the experiment, it was determined that the mean IOP in both eyes for all measurements was 16.1 ± 6.4 mmHg. The mean IOP in the right eye was 15.9 ± 5.7 mmHg (7.7 to 34.0 mmHg) and the mean IOP in the left eye was 16.4 ± 7.1 mmHg (7 to 37.7 mmHg). The median was 15.3 mmHg. The top 90 ${\;}^{th}$ percentile (P90) for both eyes was 22.1 mmHg and the bottom 10 ${\;}^{th}$ percentile (P10) was 9.0 mmHg. As represented in the box and whisker plot [Figure 1], the interquartile range (P25–P75) ranged between 12.0 and 19.6 mmHg.

**Figure 2:**
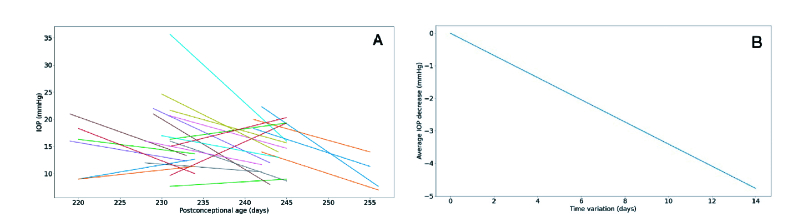
(A) The figure shows a graphic representation of IOP values variation with PCA (in days) for each of the 24 preterm infants evaluated. (B) The graph shows an average IOP decrease of 0.47 mmHg per day

Out of the 34 examined eyes, 24 had at least two IOP measurements, separated by two weeks. In this subset, the mean IOP showed an average reduction of 4.8 ± 6.7 mmHg (*P* = 0.0019) every two weeks of increased PCA, as represented in Figure 2.

##  DISCUSSION

In 1999, Ricci et al^[3]^ evaluated the IOP of 20 preterm infants and concluded that the mean IOP following birth decreased progressively. As many other studies have shown since then, our study also reports that the IOP of preterm infants decreases significantly with the increase of PCA. Normative values are not established as most studies use different tonometers and inclusion criteria. Ng et al^[4]^ reports that of all recent studies regarding IOP in premature babies, the mean/median IOP varies between 10.1 and 18.6 mmHg. In our sample, the average IOP ranged between 12 and 19.6 mmHg (P25–P75) with a median value of 15.3 mmHg. These values are in agreement with McKibbin et al^[5]^ and Axer-Siegel et al^[6]^ whose analyses obtained mean IOP values ranging between 15.5 and 16.3 mmHg.

Regarding IOP decrease with PCA, Ng et al^[4]^ reported an IOP reduction of 0.11 mmHg (*P* < 0.001) for each week increase in the PCA, however, it included evaluations of children with more than 40 weeks of age. Lindenmeyer et al^[7]^ reported a reduction of 0.29 mmHg (*P* = 0.047) per PCA week. In the current study, IOP reduction was significantly larger, 4.8 ± 6.7 mmHg (*P* = 0.0019) with every two weeks increase in PCA. First of all, this may be explained by the fact that we included extremely preterm infants with very low gestational ages who were not included in other studies. Secondly, the IOP measuring method used was also different as Tonopen II was used. Thirdly, artificial elevation of IOP may occur in infants who are awake and react with vigorous resistance to the ophthalmic examination.^[4]^

Although the theory of IOP decrease with increasing PCA seems to have a relative consensus as shown in recent studies, the exact physiopathological mechanism behind this fact is still not well established. Physiopathological mechanisms that may explain this consistent IOP decrease with increasing PCA include increasing size of ocular structures, anterior chamber angle maturation and progressive improvement of the aqueous drainage system and neuroendocrine changes. The exact mechanism is yet to be discovered.

It is well known that infants with ROP may have increased IOP values due to several treatment options such as laser and anti-VEGF therapy and changes in advanced ROP stages that may lead to angle closure. There are other proposed physiopathological mechanisms that may cause increases in IOP measurements which are not unique to ROP but are related to prematurity in itself such as incomplete trabecular meshwork development, decreased anterior chamber depth, anteriorly displaced iris planes, and increased lens thickness.^[9]^ In our study, we only included a small number of children with early stages of ROP (stage I or II). As none of them were submitted to laser or to anti-VEGF therapy that could alter IOP values, it is highly unlikely that early ROP changes would lead to significant IOP changes in these eyes and we chose to include them in the study.

Most studies regarding IOP values in premature infants included the use of an eyelid speculum and anesthetic drops for the IOP measurements which were obtained with the use of TonoPen XL ${\;}^{TM}$ .

We chose to use iCare IC200 for measuring IOP in preterm infants. This method has the advantage of not needing anesthetic drops or eyelid speculum placement. There have been studies that reported that the use of an eyelid speculum may raise IOP by an average of 4 mmHg in children.^[8]^

Haus et al^[10]^ compared the iCare IC200 rebound tonometer (ICT) with the TonoPen XL (TP) measurements in premature infants and concluded that IOP values evaluated by the ICT were significantly lower than that of the TP. Tonopen use requires anesthetic eye drop instillation and a large applanation area which implicates an increased width of the eyelid opening. This may cause discomfort to the child leading to falsely elevated IOP values. Therefore, the iCare rebound tonometer might be a better option to measure IOP in premature children. Its main advantages include the extensive flexibility in positioning with the possibility of the patient being placed in a supine position, no need to anesthetic drops and little or no discomfort for the child.

As far as we are aware, this is the first prospective longitudinal study using iCare IC200 tonometer in preterm infants. This IOP-measuring method is fast, accurate, and reliably repeatable, and thus it should be considered a first-line option for the IOP evaluation in premature children.

Our study had certain inherent limitations. On one hand, eyelid edema after birth made some of the readings hard and probably less reliable as the infant had difficulty opening one or both eyes, and manual opening of the eyelid might have resulted in inadvertent pressure on the scleral-corneal interface that might have influenced IOP measurements. On the other hand, the sample size of this study was relatively small, which may limit the statistical strength of the findings. We are also of the view that the study would have been strengthened if the measurement of corneal thickness and its correlation with IOP values were considered. Therefore, further studies with prospective, controlled design, and a larger number of included patients are needed to confirm our findings.

##  Financial Support and Sponsorship

This research did not receive any specific grant from funding agencies in the public, commercial, or not-for-profit sectors. The authors also declare they have no relevant financial or non-financial interests to disclose.

##  Conflicts of Interest

There are no competing interests.
